# Evolution of Epigenetic Mechanisms and Signatures

**DOI:** 10.3390/cells12010109

**Published:** 2022-12-27

**Authors:** Alla Kalmykova, Anton Buzdin

**Affiliations:** 1 Koltzov Institute of Developmental Biology of Russian Academy of Sciences, Moscow 119334, Russia; 2 Institute of Molecular Genetics of National Research Centre «Kurchatov Institute», Moscow 123182, Russia; 3 World-Class Research Center “Digital Biodesign and Personalized Healthcare”, Sechenov First Moscow State Medical University, Moscow 119048, Russia; 4 Moscow Institute of Physics and Technology, Dolgoprudny 141701, Russia; 5 Shemyakin-Ovchinnikov Institute of Bioorganic Chemistry, Moscow 117997, Russia; 6 PathoBiology Group, European Organization for Research and Treatment of Cancer (EORTC), 1200 Brussels, Belgium

DNA methylation, histone posttranslational modifications, higher-order chromatin organization and regulation by noncoding RNAs are considered as the basic mechanisms underlying the epigenetic memory. The Special Issue of *Cells* “Evolution of Epigenetic Mechanisms and Signatures” combines twenty articles on different aspects of epigenetic regulation. Two reports here address the impact of chromosome-specific regulatory mechanisms on whole-genome epigenetic marks. Zhuang et al. studied the sex-biased DNA methylation patterns in mammalian tissues and demonstrate the impact of both the sex phenotype and sex chromosome complement in sexual dimorphism of the epigenome [1]. In the case of Drosophila, Ekhteraei-Tousi et al. discuss the role of complex satellite repeats enriched at the X chromosome in the genome-wide effects caused by chromosome-specific alterations [2]. Specific enzymes establish major histone modification patterns. Maksimov and Koryakov studied the interplay between two Drosophila H3K9-specific histone methyltransferases and demonstrate the tissue-specific pattern of their binding to the common targets [3]. Growing evidence indicates an important epigenetic role of three-dimensional chromosome organization. George et al. address the spatial organization of chromosome territories in mosquitoes and demonstrate cell-type-specific frequencies of chromosome–nuclear envelope and interchromosomal contacts that may affect gene expression [4]. The essential breakthrough in epigenetics came with the discovery of small endogenous Piwi-interacting RNAs (piRNAs) that silence transposable elements (TEs) and regulate gene expression in animals, especially in the germline (rev. in [5]). piRNAs direct transcriptional silencing of the cognate loci through DNA or histone methylation. TE insertions in the piRNA clusters, specific genomic regions producing piRNAs, provide an adaptive immunity to TE expansion. Maupetit-Mehouas and Vaury review the mechanisms of TE reactivation observed in the germline of different species within specific developmental windows and discuss an intriguing possibility that TE derepression could indirectly increase piRNA production, which provides TE silencing later in the development [6]. Casier et al. review the role of piRNAs in transgenerational and, particularly, environmentally induced epigenetic inheritance [7]. Legoff et al. [8] also discuss the transgenerational inheritance of environmentally induced epigenetic changes in mice and other species. A focus is placed on a mechanism of transgenerational epigenetic inheritance mediated through modification and redistribution of chromatin proteins in gametes modulated by environmental factors. Komarov et al. explore the quantitative level of piRNAs required for heterochromatinization and piRNA production from cognate loci in the *Drosophila* germline [9]. They demonstrate that transcriptional silencing and piRNA production are initiated by a minimum threshold level of complementary piRNAs, indicating a high sensitivity of the piRNA-mediated defense system. Using single-molecule sequencing technology, Mohamed et al. report that new TE insertions are not preferentially trapped by piRNA clusters but rather randomly distributed in the genome of an unstable Drosophila strain [10]. Jensen et al. provide evidence on a new regulatory pathway in mosquitoes involving short genomic sequences that fuel a concerted piRNA biogenesis in the germline [11]. In their review, Gamez et al. nicely illustrate the evolution of Piwi-piRNA pathways in Dipteran and highlight a high degree of species-specific differences in the piRNA pathway between Drosophilids and mosquito Dipteran lineages [12]. 

Machnik et al. [13] concentrate on another aspect of epigenetic silencing of TEs in mammals by means of Krüppel-associated box zinc finger proteins (KRAB–ZNFs). They also discuss the current knowledge of environmental impacts on epigenetic profiles in humans; possible effects on human health; and, more generally, evolutionary adaptation. The papers contributed by Nikitin et al. [14] and Igolkina et al. [15] investigate the possibility of using TEs as the tools for quantifying the rates of regulatory evolution of human genes and molecular pathways. The authors measure contents of modified histones H3K4me1, H3K4me3, H3K9ac, H3K27ac, H3K27me3, and H3K9me3 within the relatively recently integrated TEs in comparison to non-repetitive adjacent DNA. A greater degree of migration of functional regions, such as modified histone binding sites within the TEs means their faster regulatory evolution, and vice versa [14,15]. Interestingly, histone tags showed different regulatory patterns for active chromatin vs. heterochromatin tags [15]. Fastest regulatory evolution was detected for the genes involved in the processes of gene silencing by small RNAs, DNA metabolism/chromatin structure, sensory perception/neurotransmission, and lipids metabolism. The slowest were connected with innate immunity; protein ubiquitination/degradation; cell adhesion, migration, and interaction; metal metabolism/ion transport; cell death; and intracellular signaling pathways [14]. Bylino et al [16] review the evolution of transcriptional enhancers and conclude that enhancers of multicellular eukaryotes originated from the corresponding prototypic prokaryotic regulatory elements evolving along with the gradually increasing genome size. Mammalian genomes encode tens of thousands of long non-coding RNAs (lncRNAs), which are capable of interactions with DNA, RNA, and proteins, thereby regulating gene expression in many ways. Ibragimov et al. [17] review the role of lncRNAs in the enhancer functioning. Zimmer-Bensch highlights that nearly 40% of all human lncRNAs are expressed specifically in the brain, including hundreds of human-specific lncRNAs, which makes them plausible candidates for being actors of human brain evolution [18]. Lysine acetyltransferases (KATs) can modify many proteins, including histones, and thereby epigenetically control gene expression. They are also considered as the therapeutic targets, e.g., in cancer. However, multi-site targeting of KATs and their overlapping specificities make it difficult to precisely predict the molecular effects of their inhibition. Henry et al. created cell-based and zebrafish models, which enabled them to predict biochemical consequences of a treatment by a histone acetyltransferase inhibitor C646 specific to p300 KAT activities [19]. Laptev et al. review current progress in understanding mitochondrial ribosomal RNA modification mechanisms and decode their evolutionary history [20]. Finally, Batyrev et al. reconstruct and compare DNA methylation profiles of modern and ancestral human and pre-human DNAs. Interestingly, through 3D modeling of genome organization, they identified differentially methylated loci connected with the groups of genes enriched with functions in facial anatomy and formation of the spinal column, chin, hair, and scalp [21].

The articles presented in this Special Issue clearly emphasize the diversity and intricacy of epigenetic mechanisms that are still far from being understood.
